# Induction chemotherapy with methotrexate, nimustine, and procarbazine for primary CNS lymphoma in the elderly: a retrospective evaluation of safety and efficacy

**DOI:** 10.1007/s11060-026-05435-4

**Published:** 2026-01-20

**Authors:** Yoshihiro Umezawa, Masahide Yamamoto, Keisuke Tanaka, Kota Yoshifuji, Hiroki Akiyama, Ayako Nogami, Toshikage Nagao, Takehiko Mori

**Affiliations:** 1 Department of Hematology, Graduate School of Medical and Dental Sciences, Institute of Science Tokyo, Tokyo, Japan; 2 Department of Laboratory Medicine, Graduate School of Medical and Dental Sciences, Institute of Science Tokyo, Tokyo, Japan

**Keywords:** Primary central nervous system lymphoma, Nimustine, High-dose methotrexate, Chemotherapy, Elderly patient

## Abstract

**Purpose:**

Primary central nervous system lymphoma (PCNSL) is a rare and aggressive extranodal non-Hodgkin lymphoma that predominantly affects elderly individuals. High-dose methotrexate (HD-MTX)-based chemotherapy remains the mainstay of PCNSL treatment, but the optimal combination regimen for elderly patients remains undefined. Nimustine (ACNU), a nitrosourea with excellent blood–brain barrier penetration, has not been evaluated in combination with HD-MTX. We investigated the efficacy and safety of HD-MTX combined with ACNU in elderly PCNSL patients.

**Methods:**

We retrospectively analyzed the cases of 17 untreated PCNSL patients (median age, 71 years) who received the MPA regimen consisting of HD-MTX, procarbazine, and ACNU as induction therapy. None underwent consolidation with whole-brain radiation therapy or high-dose chemotherapy with autologous stem cell transplantation. Clinical characteristics, survival outcomes, and treatment-related adverse events were evaluated.

**Results:**

The overall response rate was 100%, with complete response in 65% of the patients. The 2- and 5-year progression-free survival (PFS) rates were 67.6% and 60.1%; the 5-year overall survival rate was 73.3%. Rituximab use was significantly associated with improved PFS (*p* = 0.03); cumulative HD-MTX dose and number of ACNU cycles were not. Hematologic toxicities (neutropenia or thrombocytopenia) occurred in 59% of the patients, and hepatic toxicities in 53%. One patient died of sepsis.

**Conclusions:**

The MPA regimen yielded high response rates and durable disease control with acceptable toxicity in elderly PCNSL patients without consolidation. Rituximab use correlated with improved PFS. These findings support MPA, *particularly with rituximab*, as a promising frontline option for consolidation-ineligible patients and warrant prospective validation.

## Introduction

Primary central nervous system lymphoma (PCNSL) is a rare type of lymphoma that occurs in the brain, spinal cord, cerebrospinal fluid, or eyes, and it accounts for approx. 3% of all brain tumors [1]. Histopathologically, PCNSLs are usually identified as diffuse large B-cell lymphoma (DLBCL) and are classified as immune-privileged lymphomas in the fifth edition of the WHO Classification of Lymphomas [2]. Standard therapies for systemic DLBCL such as rituximab, cyclophosphamide, doxorubicin, and prednisolone (R-CHOP) do not sufficiently penetrate the central nervous system (CNS) and have thus led to suboptimal treatment outcomes. The current cornerstone of PCNSL treatment is high-dose methotrexate (HD-MTX) therapy, which effectively penetrates the CNS. Several research groups have described the efficacy of HD-MTX combined with an agent such as procarbazine (PCZ), vincristine (VCR), and thiotepa (TT) [3–5], but the superiority of any specific drug combination remains to be fully elucidated.

After the administration of induction therapy with an HD-MTX-containing regimen, consolidation therapies such as high-dose chemotherapy with autologous peripheral blood stem cell transplantation (HDC/ASCT) or radiotherapy (whole-brain radiation therapy [WBRT]) are often conducted as standard therapy, particularly in young patients [3, 6, 7]. In elderly patients, HDC/ASCT is highly toxic and challenging, and WBRT has been reported to increase the risk of leukoencephalopathy. It is thus critical to optimize the induction therapy for elderly patients with PCNSL by identifying agents to combine with HD-MTX that will improve the outcomes in this population without the use of consolidative HDC/ASCT or WBRT.

Nimustine (ACNU) is a nitrosourea-class antineoplastic agent that has been used in Japan for the treatment of glioma [8, 9], and ACNU was approved in Japan for the treatment of malignant lymphoma in 1979. Nitrosoureas are known for their favorable permeability across the blood–brain barrier, and agents such as carmustine (BCNU), lomustine (CCNU) and ranimustine (MCNU) have demonstrated efficacy for PCNSL when used in combination with HD-MTX [6, 10–13]. Nitrosoureas have traditionally been incorporated into intensive induction regimens for PCNSL [6, 10, 13]. Notably, prior studies have suggested that nitrosourea-containing regimens adapted for elderly patients may be feasible and effective [11, 12]. However, the efficacy and safety of combination therapy with ACNU and HD-MTX have not been evaluated. We therefore conducted the present retrospective analyses to assess the efficacy and safety of HD-MTX therapy combined with ACNU (without consolidation therapy) in elderly patients with newly diagnosed, untreated PCNSL.

## Patients and methods

### Patients

We retrospectively analyzed the cases of elderly patients with previously untreated PCNSL who had received the MPA regimen, which consists of HD-MTX, PCZ, and ACNU, as induction therapy at the Institute of Science Tokyo Hospital during the period from July 2012 through November 2020. Elderly patients were defined as those aged ≥ 60 years at the time of diagnosis*.* Each patient’s diagnosis of PCNSL was made based on a brain biopsy. In cases with both intraocular and CNS involvement, lymphoma was diagnosed based on the diagnostic criteria for vitreoretinal lymphoma, and a definitive diagnosis of PCNSL with intraocular involvement was made without performing a brain biopsy. Each patient had undergone a whole body evaluation with positron emission tomography/computed tomography (PET/CT) or CT prior to treatment. We excluded cases in which lesions were detected outside the CNS. This study was conducted in accordance with the Helsinki Declaration and was approved by the Ethics Committee of the Institute of Science Tokyo (approval no. M2000-2157).

### Treatment regimen

The patients’ MPA therapy was adapted from the French Neuro-Oncology Association protocol (HD-MTX, PCZ, and CCNU) [13], with CCNU replaced by ACNU, thus providing the formulation approved for use in Japan. This MPA regimen was implemented following approval by our hospital’s Regimen Review Committee and consisted of one or two monthly cycles of MTX, PCZ, and ACNU. Each cycle was structured as follows. MTX (3.0 g/m^2^) was infused over a 3-hr period on days 1, 11, and 21, with standard pretreatment hydration and urinary alkalinization. Leucovorin rescue was begun 24 h after the MTX infusion and continued for ≥ 72 h or until the patient’s serum MTX level fell to < 0.1 µM. PCZ was administered at 100 mg/m^2^/day for 7 consecutive days, and ACNU (40 mg/m^2^) was administered on day 1. Dose adjustments or treatment delays/discontinuation of MTX and ACNU were determined at the discretion of the treating physician. Rituximab (375 mg/m²), when administered, was given intravenously one or two times per cycle at the discretion of the treating physician. In two patients with intraocular involvement diagnosed without brain biopsy, CD20 expression could not be assessed due to limited diagnostic material, and rituximab was therefore not administered.

### Prognostic models

Two prognostic models were adopted for this analysis. The International Extranodal Lymphoma Study Group (IELSG) model incorporates five prognostic factors: age > 60 years, an Eastern Cooperative Oncology Group (ECOG) Performance Status (PS) > than 1, an elevated serum lactate dehydrogenase (LDH) level, an increased cerebrospinal fluid (CSF) protein concentration, and the involvement of deep brain structures. Each of the prognostic factors is scored as 0 points when favorable or 1 point when unfavorable, and the total score determines the risk group (0–1 points: low risk, 2–3 points: intermediate risk, 4–5 points: high risk) [14].

The Nottingham/Barcelona (NB) model is based on three risk parameters: age ≥ 60 years, ECOG PS > 1, and the presence of either multifocal lesions or meningeal involvement [15].

### Efficacy and safety assessments

The patients’ treatment responses were assessed based on gadolinium-enhanced magnetic resonance imaging (MRI) results and evaluated based on the International PCNSL Collaborative Group Response Criteria [16]. When MRI could not be performed because of a patient’s reduced activities of daily living (ADLs) or other clinical limitation, CT was used as an alternative imaging modality. Treatment-related adverse events were evaluated based on the Common Terminology Criteria for Adverse Events (CTCAE) ver. 5.0: oral mucositis, renal impairment (evaluated based on elevated creatinine), hepatic impairment (evaluated based on elevated alanine transaminase [ALT] or bilirubin), neutropenia, and thrombocytopenia. The development of infections or febrile neutropenia was also evaluated.

### Statistical analyses

The patients’ overall survival (OS) was defined as the time from the date of initial diagnosis to death from any cause or the last follow-up. Their progression-free survival (PFS) was defined as the time from the date of diagnosis to the first occurrence of disease progression, death from any cause, or the last follow-up, whichever occurred first. Disease progression was defined as recurrence or worsening within the brain parenchyma, excluding local recurrence with intraocular lesions. The OS and PFS rates were estimated using the Kaplan–Meier method and compared between groups using the log-rank test. Multivariate analyses of PFS were performed using the Cox proportional hazards model, including factors with a p-value < 0.1 in the univariate analysis. All statistical analyses were performed using EZR software (ver. 1.54; Saitama Medical Center, Jichi Medical University, Saitama, Japan) [17].

## Results

### Patient characteristics

During the study period, 26 patients with PCNSL were diagnosed at our hospital, of whom 23 were aged ≥ 60 years and classified as elderly. Among these elderly patients, six did not receive induction chemotherapy with the MPA regimen because of poor performance status and/or comorbidities, at the discretion of the treating physician. Accordingly, the remaining 17 patients received the MPA regimen and were included in the present analysis*.* Table 1 summarizes the patients’ characteristics, treatments, and efficacy. These patients consisted of seven men and ten women, with a median follow-up period of 51 months. The median age was 71 years (range, 67–80 years). Deep structure involvement was observed in 15 (88%) patients, and multifocal involvement was observed in nine (53%) patients. Fifteen patients were diagnosed based on a brain biopsy, and the remaining two patients had ocular involvement and were diagnosed based on the results of a vitreous fluid analysis.

**Table 1:** Patient characteristics, treatments, and outcomes for the study population: 17 elderly patients with primary central nervous system lymphoma (PCNSL)

Patient characteristics at diagnosis														Treatment					Outcome			
**Sex**	**Age**	**PS**	**Immunohistochemistry**			**Ocular** **involvement**	**CSF**		**Disease lesion**		**LDH elevation**	**Risk score**		**MTX**		**ACNU** **times**	**Rituximab** **times**	**MPA** **courses**	**Response**	**Relapse**	**Outcome**	**Cause of death**
			**CD10**	**CD20**	**COO**		**Cytology**	**Protein**	**Deep ** **lesion**	**Multiple lesions**		**IELSG**	**NB**	**Times**	**Total dose**,** mg/m**^**2**^							
F	68	0–1	−	+	n.a.	−	−	Elevated	+	་	−	Int	2	3	8920	1	1	1	PR	+*	A / 84.5 mos.	
F	70	0–1	−	+	non-GCB	−	−	Normal	+	−	+	High	1	5	15,040	2	2	2	CR	−	A / 112 mos.	
M	78	0–1	−	+	n.a.	−	་	Elevated	+	−	+	High	1	3	8940	1	1	1	PR	−	A / 12.3 mos.	
F	71	0–1	−	+	non-GCB	−	−	Elevated	+	−	−	Int	1	6	17,830	2	2	2	CR	−	A / 53.6 mos.	
M	71	> 1	+	+	GCB	−	་	Elevated	+	་	+	High	3	1	2040	1	1	1	PR	−	A / 2.1 mos.	
F	72	> 1	−	+	n.a.	−	n.a.	n.a.	−	−	−	n.a.	2	3	8940	1	1	1	CR	−	A / 1.9 mos.	
F	80	> 1	−	+	non-GCB	−	−	Normal	་	+	+	High	3	6	16,350	1	2	2	CR	−	A / 92.1 mos.	
F	74	0–1	n.a.	n.a.	n.a.	+	་	Elevated	−	−	−	Int	1	3	9000	1	0	1	CR	−**	A / 102 mos.	
M	71	> 1	+	+	GCB	−	−	Normal	+	+	−	Int	3	5	13,020	2	1	2	CR	+	D / 48.7 mos.	PCNSL
M	78	> 1	+	+	GCB	−	n.a.	n.a.	+	−	+	High	2	1	2950	1	1	1	PR	−	D / 1.5 mos.	Sepsis
M	78	> 1	+	+	GCB	−	་	Elevated	+	+	−	High	3	1	2850	1	0	1	PR	−	D / 1.2 mos.	MI
M	70	0–1	−	+	n.a.	−	n.a.	n.a.	+	+	−	n.a.	2	6	17,440	2	2	2	CR	− **	D / 85.7 mos.	Lung cancer
F	71	> 1	+	+	GCB	−	−	Normal	+	+	−	Int	3	6	17,020	2	4	2	CR	+	A / 57.1 mos.	
M	76	0–1	n.a.	n.a.	n.a.	+	−	Normal	+	+	−	Int	2	5	13,860	2	0	2	PR	+	D / 3.3 mos.	PCNSL
F	73	> 1	+	+	GCB	−	n.a.	Elevated	+	−	+	High	2	2	3960	1	0	1	CR	+	A / 11.3 mos.	
F	67	0–1	−	+	non-GCB	−	n.a.	n.a.	+	+	−	n.a.	2	6	17,920	2	2	2	CR	−	A / 84.3 mos.	
F	71	0–1	−	+	non-GCB	−	−	n.a.	+	−	+	n.a.	1	6	18,320	2	2	2	CR	−	A / 66.6 mos.	

*Intraocular recurrence (36 months); **Intraocular recurrence without subsequent CNS recurrence. A: Alive, CNS: central nervous system, COO: cell of origin, CR: complete response, CSF: cerebrospinal fluid, D: Dead, GCB: germinal center B-cell-like, IELSG: International Extranodal Lymphoma Study Group, Int: intermediate, LDH: lactate dehydrogenase, MI: myocardial infarction, mos.: months, MTX: methotrexate, MPA: methotrexate + procarbazine + ACNU, n.a.: not available, NB: Nottingham/Barcelona, PCNSL: primary central nervous system lymphoma, PR: partial response, PS: performance status

According to the IELSG classification, six patients were at intermediate risk and seven patients were at high risk. The NB model assigned risk scores of 1 point in five patients, 2 points in seven patients, and 3 points in five patients. In the immunohistochemical staining of brain biopsy specimens, CD10 was positive in six of 15 cases (40%), and CD20 was positive in all evaluated cases. Among the 10 cases evaluable by the Hans algorithm [18], five were classified as germinal center B-cell-like (GCB) and five were classified as non-GCB.

### Treatment

All 17 patients received corticosteroids (dexamethasone, betamethasone, or prednisolone) prior to the initiation of MPA therapy, and clinical responses were observed in eight of the patients. The number of MPA therapy courses administered was one course in eight patients and two courses in the other nine patients. The reasons for completing only one course were achievement of remission (*n* = 3), decline in ADLs (*n* = 3), infection (*n* = 1), and disease progression (*n* = 1). The median number of MTX administrations was five (range 1–6), and the median total cumulative dose was 13,020 mg/m^2^ (range 2,850–18,320 mg/m^2^). Thirteen patients received rituximab with a median administration day of day 8 (range, 3–23) in each cycle, and eight patients were administered ACNU twice. No patients received consolidation therapy with HDC/ASCT or WBRT, or any maintenance therapy.

### The MPA regimen’s efficacy and the survival outcomes

Among the 17 patients, the overall response rate (complete response [CR] + partial response [PR]) was 100%, with a CR achieved by 11 patients (65%). During the follow-up period, three patients experienced local intraocular recurrence and were treated with intravitreal MTX injection. Subsequent recurrence in the brain parenchyma occurred in four patients, including one of the patients with local intraocular occurrence.

**Fig. 1:**
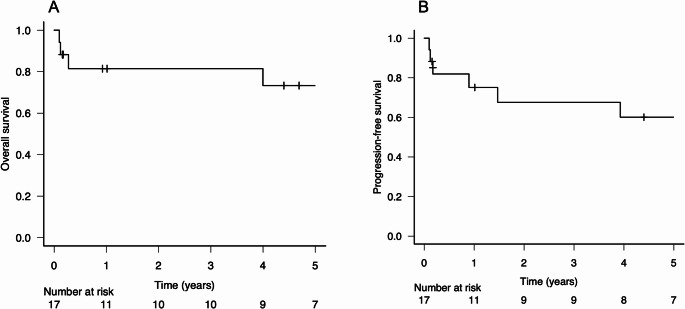
The Kaplan–Meier curves depicting (**A**) the overall survival (OS) and (**B**) progression-free survival (PFS) of the cohort of 17 elderly patients with primary central nervous system lymphoma (PCNSL)

**Fig. 2:**
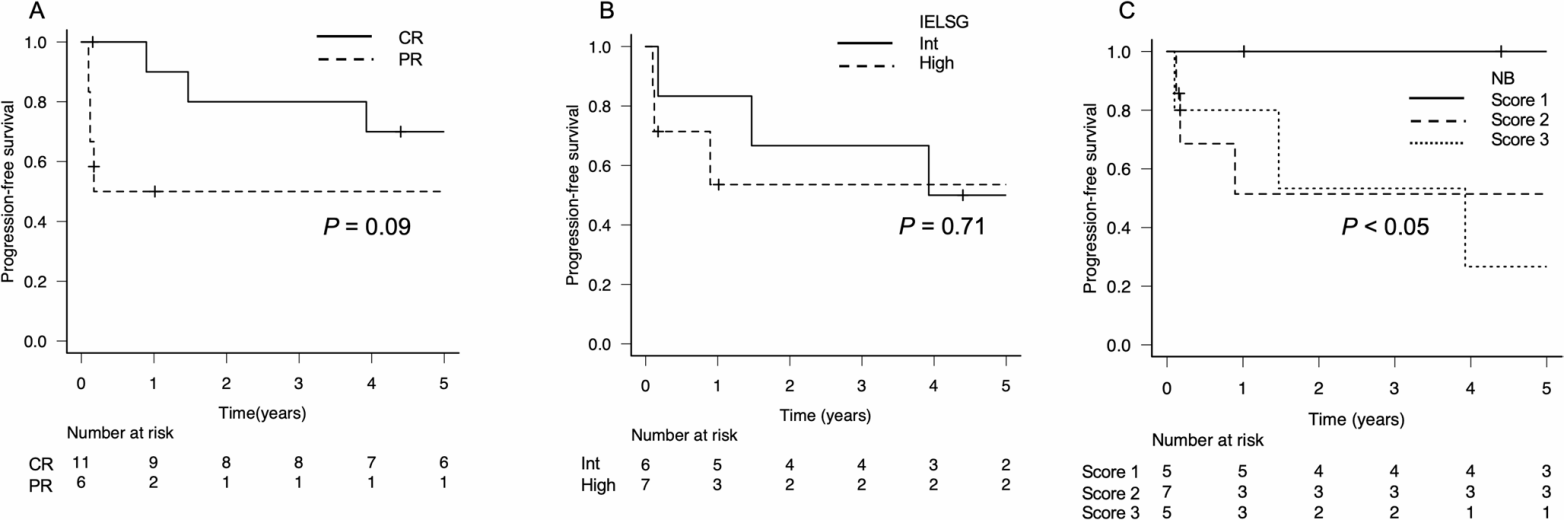
Kaplan–Meier estimates of PFS stratified by the treatment responses and the two prognostic models. (**A**) The PFS rate according to treatment response, comparing patients who achieved a complete response (CR) and those with a partial response (PR) (*p* = 0.09). (**B**) The PFS rate according to the International Extranodal Lymphoma Study Group (IELSG) risk classification, comparing intermediate-risk and high-risk groups (*p* = 0.71). (**C**) The PFS rate according to the Nottingham/Barcelona (NB) prognostic score, comparing patients with scores of 1, 2, and 3 points (Score 1 vs. Score 2 or 3: *p* < 0.05)

**Fig. 3:**
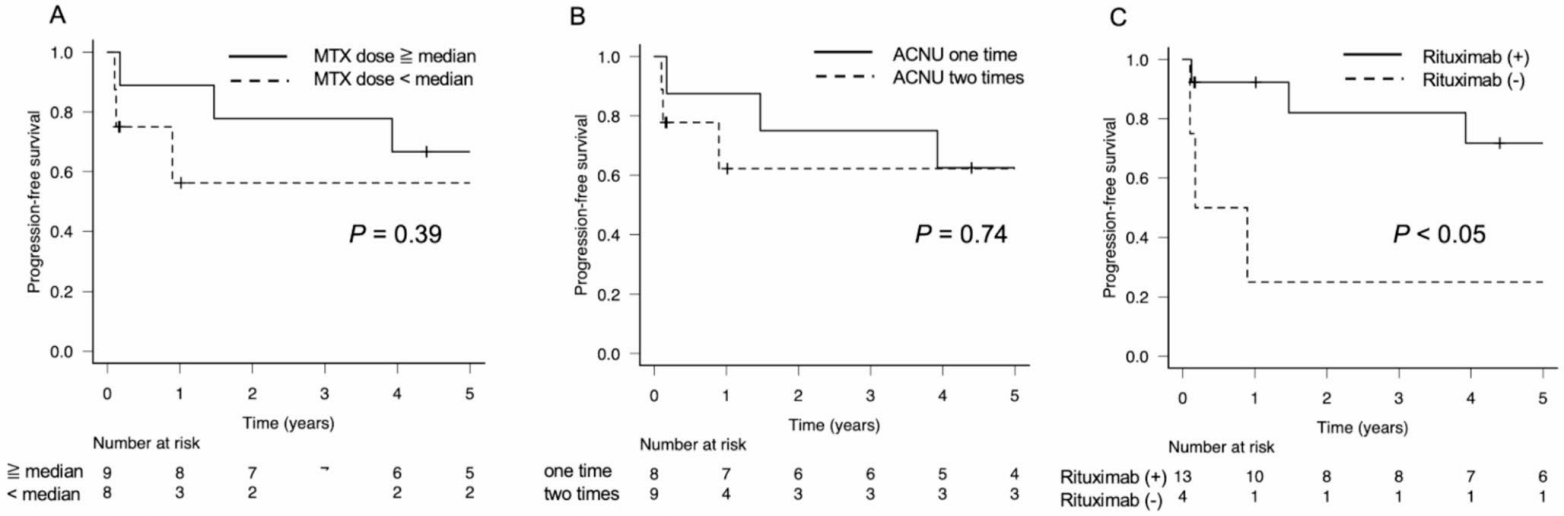
Kaplan–Meier estimates of the PFS stratified by treatment-related factors. (**A**) The PFS rate stratified by total cumulative dose of methotrexate (MTX), comparing the patients who received doses ≥ to the median vs. < the median (*p* = 0.39). (**B**) The PFS rate stratified by the number of ACNU administrations (1 vs. 2 cycles: *p* = 0.74). (**C**) The PFS rate stratified by rituximab use, comparing the patients who received rituximab and those who did not (*p* < 0.05)

For the overall patient group, the 2-year PFS and OS rates were 67.6% and 81.4%, respectively. The 5-year PFS and OS rates were 60.1% and 73.3%, respectively, although the median follow-up duration was 51 months (Fig. 1A, B). The patients who achieved a CR tended to have longer PFS periods compared to those who did not, although the difference was not significant (*p* = 0.09, Fig. 2A). In the IELSG risk classification, no difference in PFS was observed between the intermediate- and high-risk groups (Fig. 2B). In contrast, in the NB risk classification, the score 1 risk group showed a significantly better prognosis than the score 2 or 3 groups (*p* < 0.05, Fig. 2C). Further analyses based on the treatment components revealed no significant difference in PFS with respect to the total cumulative dose of MTX, using the median dose as a cutoff, or the number of ACNU administrations (Fig. 3A, B). However, a significant difference in PFS was observed between the patients who received rituximab and those who did not (*p* < 0.05, Fig. 3C). By the end of the follow-up, five patients had died, with only two of these deaths caused by lymphoma. Among the remaining three patients, one died from sepsis, one from myocardial infarction, and one from lung cancer; the latter two were considered unrelated to lymphoma.

### Safety

The adverse events that were observed are summarized in Table 2. Although renal impairment was observed in six patients (35%), no severe cases were reported. Delayed MTX excretion (defined as a serum MTX level remaining above 0.1 µmol/L at 72 h post-treatment) was observed in nine patients and in 13 of the 62 total MTX administrations (21%). Oral mucositis was noted in four patients (24%), but no clear association with delayed MTX excretion was identified. Although hepatic dysfunction occurred in nine patients (53%), a return to baseline levels after treatment was observed in all of these patients.

**Table 2:** Adverse events observed during treatment with the MPA regimen* for primary central nervous system lymphoma (PCNSL) (*n* = 17 patients)

	All grades *n* (%)	≥Grade 3 *n* (%)
Hepatic impairment	9 (53%)	5 (29%)
Renal impairment	6 (35%)	0 (0%)
Oral mucositis	4 (24%)	1 (6%)
Neutropenia	10 (59%)	6 (35%)
Thrombocytopenia	10 (59%)	4 (24%)
Febrile neutropenia	2 (12%)	2 (12%)
Infection	8 (47%)	7 (41%)

*The MPA regimen = high-dose methotrexate (HD-MTX), procarbazine (PCZ), and nimustine (ACNU)

Regarding hematological toxicity, all-grade neutropenia was observed in ten (59%) of the patients and thrombocytopenia was observed in ten (59%) of the patients, whereas grade ≥ 3 events occurred in six (35%) and four (24%) of the patients, respectively. Infectious disease developed in eight (47%) patients, and febrile neutropenia developed in two patients (12%). One patient died from sepsis early after the initiation of treatment.

### Univariate and multivariate analyses of the patients’ PFS

We used the Cox proportional hazards model to analyze the patients’ PFS, incorporating components of the NB risk model and treatment-related factors. Since all 17 patients were aged ≥ 60 years, we used their median age (71 years) as the cutoff value. The results of the univariate and multivariate analyses are presented in Table 3. In the univariate analysis, the ECOG PS and the use of rituximab were significantly associated with PFS. These two factors remained significant in the multivariate analysis.

**Table 3:** Results of the univariate and multivariate analyses of the progression-free survival (PFS) of the 17 elderly patients with primary central nervous system lymphoma (PCNSL)

	Univariate			Multivariate		
	HR	95%CI	*p*-value	HR	95%CI	*p*-value
Age, ≥ 71 vs. <71 yrs	2.55	0.66–9.85	0.17			
ECOG PS, > 1 vs. ≤1	9.79	1.12–85.65	< 0.05	13.29	1.34–131.80	< 0.05
Multiple regions, yes vs. no	1.69	0.31 − 9.26	0.55			
No. of ACNU administrations, 1× vs. 2×	1.41	0.28–7.13	0.68			
Rituximab use, no vs. yes	5.14	1.01–26.19	< 0.05	7.76	1.19–50.78	< 0.05
Total dose of MTX administration (≥ median or not)	2.04	0.39–10.56	0.39			

CI: confidence interval, ECOG PS: Eastern Cooperative Oncology Group performance status, HR: hazard ratio, MTX: methotrexate

## Discussion

The results of our retrospective analyses of the efficacy and safety of the MPA regimen, which combines HD-MTX, PCZ, and ACNU with or without rituximab, for the treatment of PCNSL in elderly patients are promising. The efficacy of polychemotherapy regimens containing HD-MTX for PCNSL has been reported by several research groups, with overall response rates (ORRs) of 70%–90% and complete response rates (CRRs) of 45%–75% [3, 7, 19–21]. Notably, the MPA regimen administered to the present patients demonstrated comparable or slightly superior efficacy, with an ORR of 100% and a CRR of 65%. Among the three patients who received only a single dose of MTX because of early treatment discontinuation due to an adverse event or a decline in ADLs, all achieved a response, but none attained a CR. In contrast, among the 14 patients who received MTX at least twice, 11 achieved a CR, indicating a remarkably favorable treatment response after induction therapy.

In addition, although our study did not include an evaluation of consolidation therapy such as HDC/ASCT or WBRT, our patients’ 2-year and 5-year PFS rates were 67.6% and 60.1%, respectively, and their 5-year OS rate was 73.3%. These long-term outcomes are comparable to those reported in studies that incorporated consolidation therapy [3, 7, 19–21]. This finding highlights the potential of the MPA regimen as an effective frontline option for elderly patients who are often poor candidates for intensive consolidation because of toxicity concerns.

Regarding the efficacy of regimens containing nitrosourea-class agents, Omuro et al. reported a best documented ORR of 78% and a CRR of 48% afforded by a regimen consisting of CCNU, HD-MTX, PCZ, and intrathecal chemotherapy [13]. In a study in which treatment with CCNU was replaced with MCNU, the ORR was 100%, the CRR was 41%, and the 2-year PFS rate was 43%, further supporting the effectiveness of regimens incorporating nitrosoureas [12]. Concerning ACNU, although its use via intra-arterial injection in combination with radiotherapy has been described [22], our search of the relevant literature identified no studies describing systemic chemotherapy combining ACNU with HD-MTX. The ACNU-containing regimen administered to the present patients demonstrated high efficacy. We thus speculate that although direct comparisons with other nitrosoureas are not feasible, this regimen with ACNU may be a highly promising therapeutic option for PCNSL in elderly patients.

The patients in this study who received only a single dose of MTX did not achieve a CR, which suggests insufficient efficacy; however, when we analyzed the PFS based on the median total MTX dose, no significant difference was observed. In contrast, the patients treated with rituximab demonstrated a notably favorable 5-year PFS rate at ~ 70%, and the multivariate analysis identified rituximab administration as a significant independent prognostic factor (Table 3). The improvement in prognosis associated with rituximab and the lack of an impact of the MTX dose on outcomes have been observed in other studies [23–25], and similar findings were confirmed with the present regimen. Moreover, the number of ACNU administrations did not affect our patients’ prognoses, suggesting that even a single dose of ACNU may suffice to achieve a therapeutic benefit.

In a report on glioma from the Japan Clinical Oncology Group (JCOG), combination therapy with ACNU and PCZ caused grade ≥ 3 neutropenia in approx. 70% of the patients [26], raising concern about potential myelosuppression with the MPA regimen. However, in the present study, the incidence of grade ≥ 3 neutropenia with the MPA regimen was only 35%. A commonly used regimen for PCNSL is R-MPV (rituximab, methotrexate, procarbazine, vincristine) followed by cytarabine, and this regimen was reported to cause grade ≥ 3 neutropenia in 30%–50% of cases [27, 28], indicating that the frequency of neutropenia that we observed with the MPA regimen is not particularly high. One of the present patients (6% of the 17 patients) died early after the initiation of treatment due to infection, but treatment-related mortality rates of 5%–7% have also been reported with other chemotherapy regimens [29], suggesting a comparable safety profile. Moreover, although our cohort consisted of patients with a median age of 71 years, all of the instances of hepatic and renal toxicities were reversible, and the incidence of delayed MTX excretion was consistent with those of earlier studies [30, 31]. These findings suggest that with appropriate supportive care and infection control, the MPA regimen can be safely administered even in elderly or clinically vulnerable populations.

This study has limitations inherent to its retrospective design, including potential selection bias and a small sample size, which reduce the statistical power to identify prognostic factors or generalize the findings. In addition, heterogeneity in patient characteristics such as variations in the ECOG PS, MTX dosing, and rituximab administration may have influenced the patients’ outcomes. Therefore, prospective multicenter studies with larger patient cohorts are warranted to more robustly assess the efficacy and safety of this approach in elderly patients with PCNSL.

In conclusion, the results of the present analyses demonstrated that the MPA regimen afforded high response rates and favorable long-term survival outcomes in elderly patients with PCNSL, without the need for consolidation therapy and with manageable toxicity. The addition of rituximab was associated with improved clinical outcomes. These findings suggest that the MPA regimen, with the incorporation of rituximab, is a promising treatment option for patients who are ineligible for intensive consolidation and warrants prospective evaluations to confirm these encouraging results.

## Data Availability

The datasets for this article are not publicly available. Requests to access the datasets should be directed to the corresponding author.
